# Resolving the Conflict between Infectious Disease and Beneficial Urban Nature

**DOI:** 10.1093/biosci/biaf207

**Published:** 2026-02-12

**Authors:** Katherine Robertson, Holly Kirk, Jacinta Humphrey, Sarah Bekessy

**Affiliations:** Interdisciplinary Conservation Science Lab, Centre for Urban Research, School of Global, Urban and Social Studies, College of Design and Social Context, RMIT University, 411 Swanston Street, Melbourne, Victoria 3000, Australia; School of Agriculture, Food & Ecosystem Sciences, Faculty of Science, The University of Melbourne, Grattan Street, Parkville, Victoria 3010, Australia; Interdisciplinary Conservation Science Lab, Centre for Urban Research, School of Global, Urban and Social Studies, College of Design and Social Context, RMIT University, 411 Swanston Street, Melbourne, Victoria 3000, Australia; School of Molecular and Life Sciences, Faculty of Science and Engineering, Curtin University, Kent Street, Bentley, Western Australia 6102, Australia; Interdisciplinary Conservation Science Lab, Centre for Urban Research, School of Global, Urban and Social Studies, College of Design and Social Context, RMIT University, 411 Swanston Street, Melbourne, Victoria 3000, Australia; School of Agriculture, Food & Ecosystem Sciences, Faculty of Science, The University of Melbourne, Grattan Street, Parkville, Victoria 3010, Australia; Interdisciplinary Conservation Science Lab, Centre for Urban Research, School of Global, Urban and Social Studies, College of Design and Social Context, RMIT University, 411 Swanston Street, Melbourne, Victoria 3000, Australia; School of Agriculture, Food & Ecosystem Sciences, Faculty of Science, The University of Melbourne, Grattan Street, Parkville, Victoria 3010, Australia

**Keywords:** urban nature, urban planning, biodiversity, infectious disease, One Health

## Abstract

Urban nature offers critical benefits for urban residents and is foundational for city livability. However, biodiversity in cities can also deliver disservices to people, including the potential to increase infectious disease risk. In the present Forum, we explore these tensions through a narrative review and qualitative synthesis, arguing that the possible benefits and risks of biodiverse urban areas need not be in conflict. We compile evidence linking urban nature features to zoonotic and vector-borne disease risk, identifying five interactions of concern and highlighting the importance of proactive management. Building on this, we outline various urban design and planning actions that can reduce potential risks for visitors while maintaining urban greening cobenefits. Discussing the issue alongside a One Health perspective, we emphasize the importance of an integrated, cross-sector approach to create resilient cities that support human, animal, and environmental well-being. We conclude that adaptive management can mitigate disease concerns while maximizing urban nature benefits.

## Urban nature and human health

Urban expansion is occurring at an unprecedented rate, with an estimated 68% of people expected to live in cities by 2050 (UN-Habitat 2022). At the same time, appreciation for the benefits of urban nature is growing worldwide (Visintin et al. 2025). Acknowledging the relationship between urban landscapes, human, and environmental health, the shift in attitude closely corresponds to key facets of the One Health perspective: A collaborative, interdisciplinary approach that aims to optimize health outcomes by recognizing the interconnectedness of animal, human, and environmental well-being (Mackenzie and Jeggo 2019, Ghai et al. 2022). In recent years, the One Health approach has gained popularity in combatting complex health issues at the human–animal–environmental interface (Ghai et al. 2022, Giacomini et al. 2025). Specifically, the potential of One Health for pandemic prevention has accelerated interest across various fields, spanning economics, ecology, epidemiology, and public health (Giacomini et al. 2025).

Urban nature encompasses the plants and nonhuman inhabitants found in urban spaces, including dedicated recreational areas such as parks and other multipurpose green spaces such as rooftop gardens and diverse streetscapes. An expanding body of evidence emphasizes the direct benefits urban nature provides to residents, including both mental (Bowler et al. 2010, Bratman et al. 2019) and physical outcomes (Browning et al. 2022, Soga and Gaston 2022, Nwana et al. 2024). Additionally, over the last decade, the need for innovative responses to climate change and biodiversity loss has driven the proliferation of nature-based solutions, a specific type of urban nature designed to address current environmental challenges (Eggermont et al. 2015, Garrard et al. 2018, Croeser et al. 2024). There is increasing awareness that integrating ecological systems within cities is not only an environmental imperative but also a valuable public health opportunity (Breed et al. 2021, Robinson et al. 2022, Plowright et al. 2024).

## Urban nature, zoonoses, and vector-borne disease

With growing enthusiasm for urban nature comes rising concern about its potential risks (Lõhmus and Balbus 2015, Aerts et al. 2018, Schell et al. 2021). Especially since the COVID-19 pandemic (Lu et al. 2021, Schell et al. 2021, Correia and Mammola 2024), there is an increasing worry that encouraging greater biodiversity in cities may have unintended, near-opposing consequences, including facilitating the spillover and spread of infectious diseases. In particular, the close human–nature connections fostered by biodiverse urban areas have raised questions about the potential risk of zoonotic and vector-borne diseases (Lõhmus and Balbus 2015, Aerts et al. 2018, Schell et al. 2021).

Together, zoonotic and vector-borne diseases represent over 60% of infectious diseases globally, representing a substantial ongoing health risk to humankind (Jones et al. 2008). As of 2025, every priority disease identified by the World Health Organization is zoonotic or vector-borne in nature (WHO 2025). These realities have heightened awareness of the potential unintended consequences that may arise from integrating nature into densely populated settings, at times fueling negative attitudes to nature across the globe (Lu et al. 2021, Correia and Mammola 2024).

## Terminology and scope

Although both zoonotic and vector-borne diseases originate in species other than humans, they are nonetheless two distinct epidemiological concepts (Mills et al. 2010). Zoonotic diseases (or zoonoses) originate in vertebrate animals and are typically transmitted directly from infected animals to humans (Millar and Moore 2006, Rahman et al. 2020). Vector-borne diseases, by contrast, are transmitted to humans primarily by blood-feeding arthropods such as fleas, mosquitoes, and ticks (Obradović et al. 2022). Although many vector-borne diseases are also zoonotic (Millar and Moore 2006, Rahman et al. 2020), the terms are not synonymous (Mills et al. 2010, Obradović et al. 2022). For clarity and accessibility to an interdisciplinary audience, we address them collectively throughout this Forum article and provide a glossary of key terms in box 1.

With this understanding in place, we compile evidence linking urban nature features to the risk of zoonotic and vector-borne diseases through a narrative review process. In line with One Health perspectives, we argue that these potential benefits and disservices need not be in conflict. Rather, proactive management through integrated urban design and planning can mitigate the risk of zoonotic and vector-borne diseases for visitors while maximizing the benefits of biodiverse urban areas. To demonstrate this, we identify five interactions of concern and outline practical urban design and planning actions for risk management (e.g., Kirk et al. 2021, Humphrey et al. 2025), many of which are feasible within existing municipal design frameworks. Although previous research has recognized the potential for urban planning to reduce disease risk (Bradley and Altizer 2007), our article is, to our knowledge, the first to outline specific design actions for infectious disease management in urban nature spaces. Finally, we conclude our assessment by contextualizing it alongside other urban disease risk factors.

Box 1. **Glossary of key terms.**

**One Health:** A collaborative, multidisciplinary approach aimed at optimizing health outcomes by recognizing the interconnectedness of humans, animals, and our shared environment.

**Urban nature:** Areas composed of natural elements within cities and towns, ranging from dedicated recreational spaces such as parks and gardens, to other green spaces such as community gardens and diverse streetscapes.

**Zoonotic disease:** A disease or infection that is transmitted naturally from vertebrate animals to humans. Zoonotic diseases are caused by a wide range of pathogens. They are classified as either emerging—newly recognized, newly evolved, or increasing in incidence or geographic range—or reemerging—increasing in incidence or geographic range following a period of decline.

**Vector-borne disease:** A disease or infection that is transmitted to humans or vertebrate animals by the bite of an infected arthropod, which acts as a vector for pathogens. Common vectors include fleas, mosquitoes, and ticks. Like zoonotic diseases, vector-borne diseases can be classified as emerging or reemerging.

**Zoonotic and vector-borne disease risk:** The likelihood or probability that an individual will contract a zoonotic or vector-borne disease.

**Pathogen spillover:** The transmission of a pathogen from one species to another, also referred to as a *spillover event* or a sp*illover infection*. It is typically used to describe novel zoonotic diseases in humans, where pathogens must overcome ecological, epidemiological, and behavioral barriers to establish infection. In instances where there is no adaptation for subsequent human-to-human transmission, it may be referred to as a *dead-end spillover*.

**Urban scavengers:** Animals that feed on dead and decaying matter in urban environments. They appear well adapted to the challenges of urban centers.

**Pathogen reservoir:** The natural host where a pathogen lives and multiplies, from which it can then be transmitted to other susceptible hosts. Although pathogen reservoirs can also be environmental hosts such as soil and water, in this article we are generally discussing living organisms such as vertebrate animals.

**Pathogen intermediary:** Although pathogens may be transmitted directly from pathogen reservoirs to recipient hosts such as humans, they also occasionally involve a pathogen intermediary or bridging host, which facilitates the onward transmission to other species.

**Pathogen spillback:** The reverse of pathogen spillover, wherein a pathogen is transmitted from a novel host to the original host. Pathogen spillback can have severe consequences for wildlife populations and human health, with the possibility of reintroducing or amplifying pathogens.

## Surveying the literature

To explore the risk of zoonotic and vector-borne diseases within an urban nature context, we compiled peer-reviewed literature via a narrative review. A narrative review was selected because of the novelty of the field and accessibility across disciplines (Siddharth and Gayatri 2021), as well as our aim to extend current understandings (Greenhalgh et al. 2018); it is not intended to be exhaustive. The process involved searches of two multidisciplinary research databases, *Google Scholar* and *Web of Science*, undertaken between March and June 2025. We used a series of search strings combining key terms with Boolean operators, including *zoonotic, zoonosis, zoonoses, vector-borne, urbanisation, urban nature, urban parks, urban greening, urban, suburban, peri-urban, greenspace, green space, green infrastructure, rewilding, nature-based solutions, everyday nature*, and *nature in cities*. Broader organismal and disease terms, such as *biodiversity* and *disease* were excluded because of practical constraints, returning results into the hundreds of thousands of papers. Articles were included if they demonstrated substantial conceptual engagement with the risk of zoonotic and vector-borne diseases in relation to urban nature features, whereas those that did not meet these criteria were excluded, along with articles in languages other than English. To ensure the review used up-to-date research, papers published prior to 2000 were excluded.

In addition to database searches, we reviewed the reference lists of several key Q1 papers (e.g., Bradley and Altizer 2007, Lõhmus and Balbus 2015, Hassell et al. 2017, Combs et al. 2022, Dobigny and Morand 2022) to identify additional sources of conceptual relevance. In total, 98 Q1 and Q2 articles were considered, spanning the fields of epidemiology, urban ecology, infectious disease ecology, urban design and planning, and public health. A qualitative synthesis was employed to integrate current knowledge, evaluating the extent to which urban planning and design considerations are presently integrated into zoonotic and vector-borne disease research and identifying opportunities for future interventions.

## Zoonotic and vector-borne disease risk in urban environments

Urban environments are uniquely positioned within the conversation about the risk of zoonotic and vector-borne diseases (Bradley and Altizer 2007, Hassell et al. 2017, Combs et al. 2022, Dobigny and Morand 2022). In these contexts, dense human populations regularly coexist with other animals and their associated industries, increasing the likelihood of pathogen spillover (Hassell et al. 2017). Although the full assemblage of species in urban areas remains closely tied to local context, there is evidence of increasing biotic homogenization (McKinney 2006, Chien 2025) and a growing presence of common pathogen reservoirs within our cities, often represented by highly adaptable species (Mackenstedt et al. 2015, Schell et al. 2021). Moreover, many urban-dwelling animals exhibit heightened stress from decreased resource availability, resulting in greater viral shedding (Plowright et al. 2021). These factors, combined with the rapid movement of people throughout urban landscapes, can result in a biological cauldron of sorts within urban centers, amplifying both the risk and scale of zoonotic and vector-borne disease events (Ali and Keil 2009).

The relationship between urban settings and zoonotic and vector-borne diseases is not new (Mishra et al. 2021, Piret and Boivin 2021). Although the emergence of zoonotic and vector-borne diseases is typically framed as a rural or frontier issue, linked to deforestation and agricultural expansion (Daszak et al. 2001, Gottdenker et al. 2014, Plowright et al. 2021), history demonstrates the capacity for these diseases to emerge and flourish in urban environments (Piret and Boivin 2021). The bubonic plague began in human settlements before proliferating rapidly and widely outward (Bramanti et al. 2016); more recently, epidemics such as SARS-CoV-1 and West Nile virus have reemerged and spread within urban centers (Daszak et al. 2001, Schlundt et al. 2004). Indeed, during the COVID-19 pandemic, the connectivity to urban areas was typically responsible for transmitting SARS-CoV-2 to rural residents (Polo et al. 2022).

## Urban nature and interactions of concern

Ecological and epidemiological theory indicates that urban nature may pose a potential zoonotic and vector-borne disease threat for human populations by fostering closer human–nature proximity (Lõhmus and Balbus 2015, Aerts et al. 2018, Schell et al. 2021). Through our compilation of current literature, we identified five key areas (or interactions of concern) that are most likely to be of relevance to zoonotic and vector-borne diseases within an urban context. Although many of the interactions discussed below also offer benefits to humans, we concentrate here solely on their disservices.

### Domesticated and companion animal interfaces

Domesticated animals such as cats and dogs pose a distinct disease threat within urban ecosystems (Rahman et al. 2020, Jevtic and Savic 2021, Schell et al. 2021, Dobigny and Morand 2022). Both species are known carriers of several common zoonotic pathogens (e.g., *Campylobacter* and *Giardia*; Rahman et al. 2020) and are simultaneously deeply embedded in the urban fabric. With companion animal populations reaching into the millions in several cities (Deplazes et al. 2004, 2011), their frequent and intimate contact with green spaces represents a substantial disease risk. In particular, defecation or fouling by companion animals in natural spaces can contribute to pathogen accumulation in the soil of commonly used areas (Deplazes et al. 2011, Morgan et al. 2013).

Arguably the most well researched of these pathogens are roundworms (*Toxocara* spp.), a parasite capable of causing mild to severe infection in humans if ingested (Morgan et al. 2013). Strong evidence links the presence of cats and dogs to roundworm infections; large-scale analyses suggest a fifth of the world's parks are likely to have roundworm eggs present, with cats and dogs representing a significant source of contamination (Morgan et al. 2013, Fakhri et al. 2018). Since exposure to contaminated soil is the primary mode of transmission for human infection—especially for children under four because of geophagic behavior (Deplazes et al. 2011, Manini et al. 2012)—pathogen biocontamination is a key zoonotic risk factor for many parks and other forms of green space. Alongside roundworms, other pathogens and their infections implicated with the presence of companion animals include but are not limited to *Giardia* spp. (giardiasis or beaver fever), *Cryptosporidium* spp. (crypto), *Cystoisospora* spp. (coccidia), *Campylobacter* spp. (food poisoning), and *Salmonella* spp. (salmonella; Rahim et al. 2018).

### Urban scavengers and resource attraction

The attraction of urban scavengers such as foxes, raccoons, and rodents, pose another potential risk of zoonotic and vector-borne diseases for natural areas such as parks and gardens (Bradley and Altizer 2007, Mackenstedt et al. 2015, Combs et al. 2022). Typically attracted to unintentional food resources such as uncovered rubbish bins (Feng and Himsworth 2014, Walsh 2014, Mackenstedt et al. 2015, Schell et al. 2021), scavengers seek out these scraps in the absence of natural sustenance, decreasing the proximity between humans and pathogen reservoirs (Deplazes et al. 2004, Feng and Himsworth 2014, Plumer et al. 2014). For rodents especially, improperly stored or disposed of organic waste constitutes their most significant food resource within urban environments (Feng and Himsworth 2014). Because rodents are the most common mammalian reservoir host for zoonotic pathogens according to recent analyses, these shared vicinities due to resource attraction are particularly concerning (Johnson et al. 2020). Both the spread of hantavirus and leptospirosis are linked to the presence of rodents (Schlundt et al. 2004), and they remain a species of priority management in highly urbanized settings such as New York City (Combs et al. 2022).

Other scavenger species such as foxes are also of increasing concern for urban centers, with population densities in these settings commonly exceeding those of open landscapes (Plumer et al. 2014). Behaviorally flexible, foxes have the capacity to rapidly disperse throughout urban areas and are carriers of highly dangerous infections such as rabies and *Echinococcus multilocularis* (Deplazes et al. 2004, Schlundt et al. 2004, Plumer et al. 2014). Additionally, foxes have the potential to serve as ecological disrupters in urban ecosystems, harassing and injuring native fauna and potentially acting as pathogen intermediaries (Plumer et al. 2014). By actively predating on other vertebrate species, they promote increased cross-species contact and the potential spillback of pathogens.

### Wildlife encroachment and habitat adaptation

In addition to animals expected in urban settings, wildlife species—those not typically associated with urban settings—may also be drawn to biodiverse urban areas (Lindahl and Grace 2015, Plowright et al. 2021). Evidence suggests that an increasing number of medium-size carnivores are visiting urban centers, primarily because of habitat destruction and decreased resource availability (Bateman and Fleming 2012, Schell et al. 2021). For city dwellers, wildlife encroachment signifies a potential risk of zoonotic and vector-borne diseases because of the possible introduction of zoonotic pathogens to human populations (Bradley and Altizer 2007, Bateman and Fleming 2012). Dingoes, for instance, carry a range of common zoonoses and are increasingly found in Australian cities, attracted by large prey populations and refuge opportunities (Allen et al. 2013). Furthermore, epidemics of Ebola are frequently linked to the handling of great apes (Schlundt et al. 2004), many of which now routinely engage in crop raids at the edge of human settlements (Gilbert and Kalan 2025).

Also of significance to disease risk is the increased stress experienced by many urban wildlife populations, which may result in greater viral shedding (Faust et al. 2018, Plowright et al. 2021). Bats are a commonly cited example of this phenomenon, where nutritional stress is linked to infection and heightened shedding of the Hendra virus (Faust et al. 2018, Eby et al. 2023). However, research suggests this pattern is also replicated in other mammals, such as bovids (Ezenwa 2004, Venegas-Vargas et al. 2016). Notably, the high risk of zoonotic and vector-borne diseases associated with land-use change is in part due to the stressors exerted on local species, such as decreased food and habitat availability (Faust et al. 2018, Plowright et al. 2021). The disease hazard represented by wildlife encroachment is therefore twofold—both through the introduction of pathogens and the potential for greater viral loads.

### Vector amplification

Perhaps the most well-recognized disease risk represented by biodiverse urban areas is vector amplification. Concern of vector-borne diseases is a burgeoning global priority, especially in light of new exposures and altered transmission dynamics due to climate change (Ryan et al. 2019, Villena et al. 2024). Mosquitoes, a vector with potential to harbor diseases such as malaria, dengue fever, and West Nile virus, are of particular concern (Kache et al. 2022). Since mosquitoes require standing water to breed, they may be drawn to green space features such as shallow ponds or lakes (Kache et al. 2022), where they can then proliferate rapidly because of the absence of aquatic predators (LaDeau et al. 2015, Zhao et al. 2020).

Ticks are another vector of interest within urban ecosystems (Rizzoli et al. 2014, Lõhmus and Balbus 2015, Mackenstedt et al. 2015, Combs et al. 2022). favoring fragmented habitats, ticks have been detected in various parks and other vegetated areas used by people (Lõhmus and Balbus 2015, Yuan et al. 2020). Like mosquitoes, ticks are worrisome because of their potential for transmitting diseases with lifelong consequences, including Lyme disease (Institute of Medicine 2011). The tick burden also tends to be higher in parks more frequented by large wildlife (Bellato et al. 2021), suggesting the risk of vector-borne diseases may be related to the encroachment of mammals and other species.

### Human behaviors that increase risk

Finally, various human behaviors within urban nature areas can serve to increase potential risk of zoonotic and vector-borne diseases. Failure to pick up pets’ feces, improperly disposing of food waste, and a childhood tendency for geophagy are three examples of this, the consequences of which are explored above. Outside of these, direct supplemental hand-feeding is another behavior of concern, encouraging the unnaturally dense congregation of animals near people (Gottdenker et al. 2014, Sorensen et al. 2014). Other forms of supplemental feeding, such as urban bird feeders, can increase the risk of contracting bacterial diseases such as mycoplasmal conjunctivitis (Daszak et al. 2001). Although few studies have examined the potential disease risk posed by supplemental feeding to humans, it appears a primary source of transmission within wildlife populations (Sorensen et al. 2014, Schell et al. 2021).

Unintentional contaminations by humans, biological or otherwise, can likewise increase the potential hazards of zoonotic and vector-borne diseases (Daszak et al. 2001, Bradley and Altizer 2007, Lõhmus and Balbus 2015). This includes both environmental contamination with heavy metals—linked to immunosuppressive properties and persistent infections (Mitra et al. 2022, Zhang et al. 2024)—and the inadvertent direct introduction of pathogens. The latter can occur via the translocation of ill-advised species or through unforeseen passengers that accompany urban greening efforts (Lõhmus and Balbus 2015, Mishra et al. 2021). The introduction of small mammals, for instance, can lead to the emergence of tick-borne diseases previously unknown to that region (Rizzoli et al. 2014), and it is conceivable that the movement of certain resources within biodiverse urban areas (e.g., untreated soil) may also facilitate zoonotic introductions.

## Balancing the benefits and risks of urban nature

Although the risks of zoonotic and vector-borne diseases associated with urban nature undoubtedly warrant consideration, focusing on these alone offers an incomplete picture; diverse ecological communities also provide a range of well-documented benefits essential to sustainable cities. Termed *ecosystem services*, these range from climate and weather mitigation (Burkhard et al. 2012, Keesstra et al. 2018) to a plethora of positive human health outcomes (Kondo et al. 2018, Browning et al. 2022, Soga and Gaston 2022). Specifically, urban nature is linked with improved cardiovascular health (Nwana et al. 2024), reduced respiratory illness and allergies (Hanski et al. 2012), increased childhood bone density (Sleurs et al. 2024), and reduced prevalence of diabetes, hypertension and cardiovascular diseases (Astell-Burt and Feng 2020), as well as enhanced mental well-being (Bratman et al. 2019). Within the context of disease risk, biodiverse urban areas can promote immune regulation and reduce illness severity by encouraging diverse microbiota (Yeoh et al. 2021, Soga and Gaston 2022, Robinson et al. 2024). These combined effects mean that, overall, urban nature has the potential to deliver healthier human populations that are more resilient to disease emergence.

Acknowledging these benefits is essential within any dialogue about urban nature and potential risk of zoonotic and vector-borne diseases, because they contextualize the difficulty of managing this emerging concern. As is demonstrated by One Health perspectives, urban nature plays a critical role in the future livability of urban residents. Therefore, removing biodiversity from our cities is neither a viable nor a necessary option. Potential disease risks should not be framed as reasons to underinvest in urban nature but, rather, as opportunities to implement proactive management through urban design and planning interventions, promoting health across the human–animal–environmental interface.

Box 2: **Urban design and planning interventions to mitigate the risk of zoonotic and vector-borne diseases in an urban nature area.**

**Figure 1.**
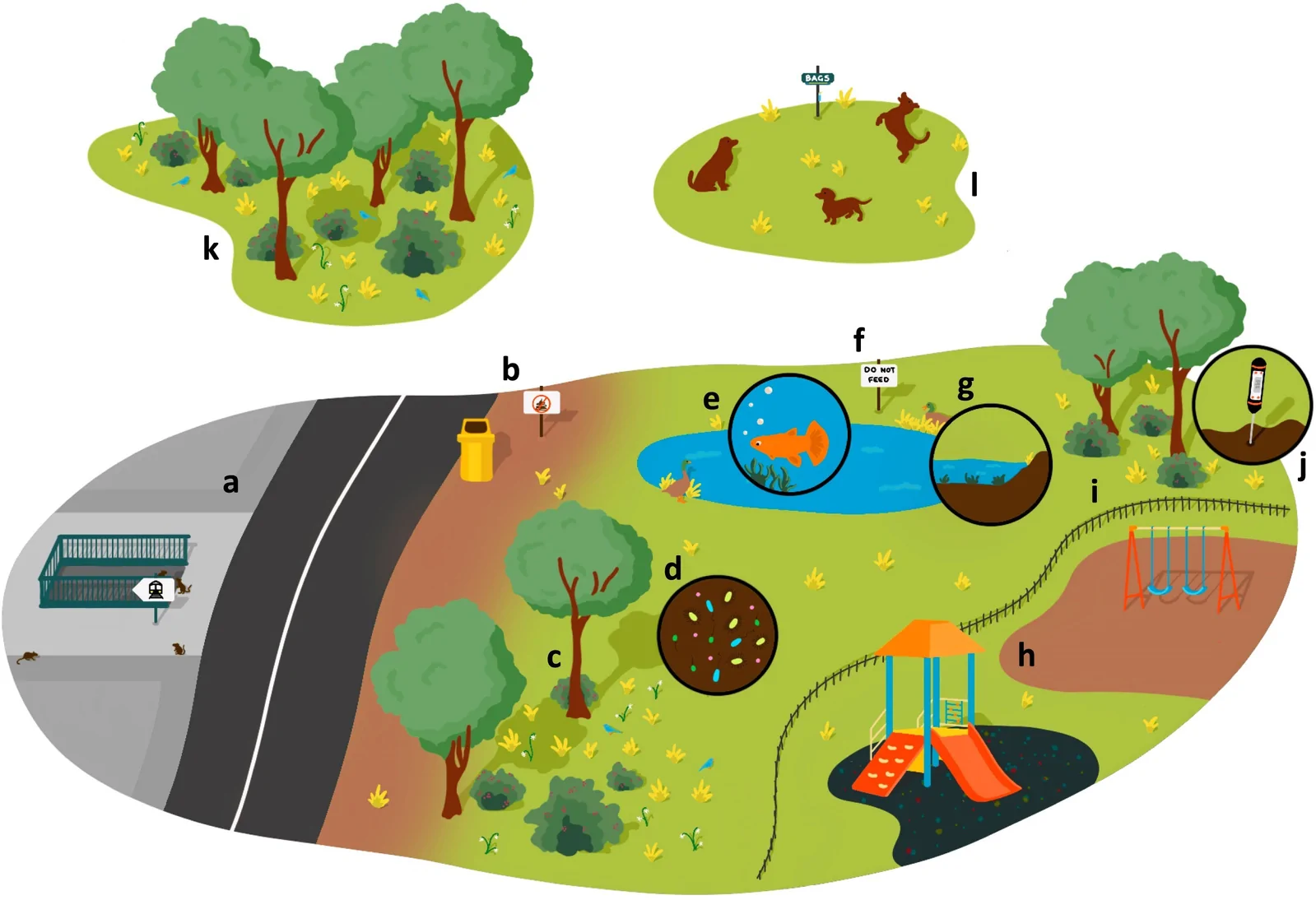
(a) Strategic placement of artificial surfaces. (b) Adequate garbage management, and waste disposal signage. (c) Biodiverse vegetation. (c) Diverse soil microbiota. (e) Diverse vertebrates in water bodies, including predators. Appropriate control of internal and external vegetation. (f) Signage discouraging the feeding of fish, ducks, and other wildlife. (g) Deep water bodies and adequate flood mitigation. (h) Use of mulch or rubber underneath play equipment. (i) Fencing around children’s play areas. (j) Testing and management of heavy metal pollutants. (k) Healthy source habitat and strategic provisioning of resources. (l) Separately designated off-leash dog areas.


**Strategic placement of artificial surfaces**. Artificial surfaces such as roads can act as barriers to the dispersal of urban scavengers and encroaching wildlife. In particular, they are observed to discourage the movement of rodents. By strategically placing them between urban nature areas and favored rodent habitats such as subways, artificial surfaces can limit rodent access to urban nature and its resources—deterring both human–rodent interactions and the sudden expansion of rodent populations. Research also suggests artificial surfaces can deter the movement of foxes and result in lower tick abundances within urban green space (Feng and Himsworth 2014, Walsh 2014, Lõhmus and Balbus 2015, Heylen et al. 2019, Tolhurst et al. 2020).

**Adequate garbage management and waste disposal signage**. Because garbage attracts urban scavengers and encroaching wildlife, adequate waste management is important to prevent accumulation that would further encourage their visitation. Educating the public on the need to clean up their pets’ feces through adequate and well-designed signage can also help limit the presence of zoonotic pathogens in the soil. Intentionally locating these at common locations for dog fouling, such as park entrances, may also be of benefit (Bradley and Altizer 2007, Deplazes et al. 2011, Walsh 2014, Lõhmus and Balbus 2015, Mizgajska-Wiktor et al. 2017, Rahim et al. 2018, Combs et al. 2022, Keegan et al. 2025).

**Biodiverse vegetation and habitat quality**. Although the relationship between biodiversity and zoonotic risk is complex and multifaceted, low biodiversity and poor habitat quality are typically associated with the persistence of urban scavenger species with fast life histories, such as foxes, raccoons, and rodents. Because these same species are also the most likely to host zoonotic pathogens, biodiverse urban nature can be seen as protective by reducing the niches available for exploitation. Ideally, vegetation should beappropriate for the area and selected with native wildlife populations in mind, aiming to limit stress and curb pathogen shedding. We also suggest considering the species of concern within vegetation decisions; for example, if rodents are a zoonotic priority, densely planted understories with evergreens should be avoided (Lõhmus and Balbus 2015, Rohr et al. 2019, Keesing and Ostfeld 2021).

**Diverse soil microbiota**. Emerging research suggests diverse soil microbiota can upregulate immune responses in humans and other species. Encouraging diversity in soils may therefore be protective against disease emergence and spread, providing a twofold benefit for public and environmental health (Robinson et al. 2024).

**Diverse vertebrates in water bodies, including predators; appropriate control of internal and external vegetation**. Water bodies represent a potential risk of zoonotic and vector-borne diseases through their ability to attract and support vector populations such as mosquitoes and vertebrate pests such as rodents. Therefore, they should be designed with these factors in mind, promoting diverse vertebrate and invertebrate communities with appropriate design of vegetation around and within the water body. For mosquitoes, the presence of predators can aid in their control, but these populations also require carefully selected aquatic vegetation: Overgrowth and low dissolved oxygen levels in the water can affect survival. Dense emergent vegetation on pond peripheries may encourage both mosquitoes and rodents, requiring regular maintenance. In general, locally native plant species should be favored, because invasive plant species are linked with an increased prevalence of mosquito-borne diseases (Lõhmus and Balbus 2015, Zhao et al. 2020).

**Signage discouraging the feeding of fish, ducks, and other wildlife**. Direct supplemental hand-feeding is a possibly harmful interaction, often bringing humans into closer proximity to dense congregations of other species. Although supplemental feeding for conservation purposes can bolster wildlife body condition and therefore reduce pathogen susceptibility, evidence indicates that feeding by private citizens most frequently achieves the opposite: instead increasing wildlife stress, disease transmission, and pathogen prevalence. Clear signage outlining the risks to both animal and human health, as well as discouraging hand-feeding, may assist in managing this concern (Gottdenker et al. 2014, Sorensen et al. 2014, Murray et al. 2016).

**Deep water bodies and adequate flood mitigation**. Dense mosquito populations are a primary zoonotic concern associated with ponds and lakes. However, the incorporation of deep-water zones may assist with mosquito control while reducing urban heat island effects—also associated with increased vector presence. Furthermore, adequate flood mitigation can assist with managing the risk of mosquito-borne diseases, alongside other conditions such as leptospirosis (Diemont 2006, LaDeau et al. 2015, Combs et al. 2022).

**Use of rubber or mulch underneath play equipment**. Although sand remains a commonly used playground surface, it is also an ideal material for animal defecation, particularly for cats. Therefore, placing an alternative material (e.g., rubber or mulch) underneath play equipment may help control this potential risk. Rubber is well suited for this purpose; however, it can also increase urban heat island effects and leach microplastics into the environment. For these reasons, we suggest using it sparingly and solely underneath play equipment. Although mulch is another option with lesser environmental impacts, it is worth noting that some animals may still seek to defecate in finer chipped varieties (Deplazes et al. 2011, Kleine et al. 2017, Mizgajska-Wiktor et al. 2017, Pacheco-Ortega et al. 2019).

**Fencing around children’s play areas**. Soil biocontamination in children’s play areas is of substantial concern. Research indicates that high fencing may assist in mitigating this potential risk, deterring the presence of domestic pets and other species. However, education on the risks of biocontamination may also be necessary to improve owner compliance, particularly in the case of dog fouling (Kleine et al. 2017, Yuan et al. 2020, Keegan et al. 2025).

**Testing and management of heavy metal pollutants**. Emerging research suggests that environmental exposure to heavy metal pollutants may exert an immunosuppressant effect on humans and animals. Ideally, soil should be tested prior to the establishment of urban nature areas to protect against this possibility. However, semiregular soil testing within current systems may also identify potential contaminate risks, after which appropriate action can be taken (Mitra et al. 2022, Zhang et al. 2024).

**Healthy source habitat and strategic provisioning of resources**. Recent evidence highlights the growing encroachment of wildlife into urban areas. In many cases, this appears linked to land-use change and destruction of their source habitat. One approach to limiting this potentially harmful behavior is by maintaining healthy source habitats outside of urban areas—including any necessary ecological corridors—or through strategic resource provisioning. For ecology professionals, this is akin to concepts of intercept feeding, wherein resources are strategically placed to lure animals away from undesired areas for their own safety (e.g., busy highways). By safeguarding core habitats or providing alternative resources elsewhere, councils can redirect encroaching wildlife while also potentially alleviating stress and pathogen shedding in local populations (Bateman and Fleming 2012, Sorensen et al. 2014, Lindahl and Grace 2015, Plowright et al. 2021).

**Separately designated off-leash dog areas**. Given the contribution of dogs to biocontamination, councils may consider designating separate off-leash areas for their use away from standing water and children’s play areas. By redirecting a portion of domestic canines and their owners, the pathogen load may be lessened in other natural areas. Provision of dog waste bags and sanitation stations in off-leash areas can also reduce zoonotic disease risks associated with frequent dog fouling (Rahim et al. 2018).

## The role of urban design and planning

The history of urban design and planning is closely intertwined with zoonotic and vector-borne diseases. In the early Middle Ages, zoonoses contributed to city planning initiatives via the construction of quarantine colonies outside nearby settlements (Davis 2022). In the 1850s, the development of sewage networks in London was primarily driven by concerns of odor and resulting disease (Davis 2022), whereas in 1894, following a bubonic plague outbreak, Hong Kong adopted strict planning and occupancy regulations for tenement blocks (Pryor 1975). In these examples, adaptive design and planning was strategically employed to mitigate disease risk—a perspective yet to be meaningfully leveraged when designing for urban nature.

As in other spaces, intentional design and planning within urban nature spaces can mitigate disease concerns; although some design elements can serve to increase potential zoonotic risk, others can serve to decrease it. Consider the case of tick distribution: Although ticks are detected in many public areas frequented by humans, their presence within school yards is at times too low to be detected, suggesting that fences excluding large wildlife may be protective against tick-borne zoonoses (Yuan et al. 2020). Likewise, reducing receptacles capable of collecting stagnant water in urban nature (e.g., plant pots or old tires) can help limit the breeding of mosquitoes (Krystosik et al. 2020). These are just two examples of many; in box 2, we outline a range of possible design and planning actions for municipal councils concerned with the risk of zoonotic and vector-borne diseases.

## Urban nature and zoonotic and vector-borne disease: What is the risk, really?

For as long as humans have lived in urban areas, animals and their corresponding diseases have coexisted in these spaces too. Records as far back as the Ancient Egyptians reveal the presence of scavengers in human settlements, and this proximity inevitably creates opportunities for zoonotic spillover (Soulsbury and White 2015). However, it is notable that when considering the source of past pandemics, urban nature is rarely mentioned as a risk factor. Instead, it is dwarfed by other human practices such as wet markets, global wildlife trade, and meat processing—all of which are more substantial disease drivers within urban settings (Schlundt et al. 2004, Bradley and Altizer 2007, Hilderink and De Winter 2021, Mishra et al. 2021, Dobigny and Morand 2022). The emergence of both SARS-CoV-1 and SARS-CoV-2 remains linked to wet markets (Hao et al. 2022), and global wildlife trade is now a multimillion-dollar business (Hilderink and De Winter 2021). As much as it is worth recognizing the potential disease risks in urban nature, it is equally important to contextualize this risk alongside other urban factors.

Moreover, in comparison to these larger-scale drivers, urban nature offers a unique opportunity for disease risks to be recognized and mitigated. Unlike other zoonotic and vector-borne disease risk factors—such as occupational exposure, which may only affect specific groups—urban nature involves a broad and diverse userbase, where potential risks emerge through visible, everyday interactions. In such settings, human–nature coexistence is both expected and encouraged, creating a distinct opportunity for disease risks to be acknowledged and proactively managed across the wider population. The above suggestions demonstrate the capacity to manage these risks through disease informed, nature-positive urban landscape design, many of which can be implemented at a municipal council level. By adopting a more integrated approach to disease risk, urban designers and planners can facilitate a responsible and safe engagement with urban nature, without extensive overhauls to current frameworks or procedures.

## Limitations of synthesis scope and proposed interventions

Although this synthesis addresses the various facets of the risk of zoonotic and vector-borne diseases in urban nature areas, it is important to note several limitations in both the scope and proposed interventions. Much of the discussion centers on the global north, reflecting broader trends in published research, which often favors advanced economies and high-income countries (Bol et al. 2023, Soga and Gaston 2023, Collyer 2024). Socioeconomic factors, including housing and sanitation standards, are significant contributors to the risk of zoonotic and vector-borne diseases (Walsh 2014, Schell et al. 2021, Combs et al. 2022, Kache et al. 2022, Villena et al. 2024), but a discussion of these falls outside the scope of this Forum article. Likewise, differing access to urban nature across geographic and demographic settings (Schell et al. 2021, Soga and Gaston 2022, 2023) and neighborhood gentrification can also affect the potential for exposure, particularly among marginalized groups (Halsey et al. 2022). For this article, our focus was on possible mitigations that can be applied in and around biodiverse areas to protect visitors to those spaces, although we acknowledge these greening actions may also interact with other complex factors to influence the risk of zoonotic and vector-borne diseases. We consider that some of the proposed interventions, such as the addition of informative signage and fencing around children’s play areas, may be adaptable across varied socioeconomic contexts.

We also note that many of the interventions described are relatively novel as mitigation strategies in the infectious disease field, where common urban landscape designs are leveraged specifically for zoonotic and vector-borne disease prevention. As a result, there is a general lack of established case studies to reference in this unique context. Where evidence does exist, we have provided links to relevant examples and their potential impact on the risk of zoonotic and vector-borne diseases in box 2. Some actions, such as the use of rubber or mulch beneath play equipment, are more widespread and typically adopted for reasons unrelated to infectious disease (Curtis et al. 2023). Signage aimed at influencing human behavior is an everyday feature of many urban spaces and, when they are well designed and appropriately placed, can effectively shape outdoor behavior (Kusmanoff et al. 2022, Rice et al. 2023, Yang 2024). In these instances, we emphasize the potential benefits of the landscape design for the risk of zoonotic and vector-borne diseases and propose their application in this lesser-used context. By contrast, other actions—such as promoting diverse soil microbiota—represent emerging areas of research that require further investigation. Where evidence is limited, our intention is to highlight the potential cobenefits these interventions might offer in reducing the risk of zoonotic and vector-borne diseases. By presenting a range of strategies, we seek to emphasize the capacity of existing urban design and management actions to contribute to mitigate the risk of zoonotic and vector-borne diseases. Nonetheless, continued evaluation, adaptation, and interdisciplinary collaboration will be essential for refining these interventions and strengthening the future evidence base.

## Conclusions

Like many issues at the forefront of current environmental challenges, urban nature embodies a complex web of nuanced and often competing relationships (Aerts et al. 2018). On one hand, it provides a multitude of benefits for humans and nonhumans alike (Bowler et al. 2010, Kondo et al. 2018, Browning et al. 2022, Nwana et al. 2024). On the other, it raises concerns about potential infectious disease emergence (Lõhmus and Balbus 2015, Aerts et al. 2018). Throughout this article, we have argued for an integrated approach toward these two perspectives, presenting clear, actionable strategies to address risks through adaptive urban design and planning. Future research may wish to expand on these suggestions by exploring the relationship between urban nature features and the risk of zoonotic and vector-borne diseases in underrepresented regions, as well as the role of community education in mitigating risks associated with domesticated animals (e.g., dog fouling) and direct supplemental hand-feeding. Answering these questions is vital to the future of healthy urban ecosystems, because halting greening policies remains an unviable and undesirable solution (Browning et al. 2022, Soga and Gaston 2023). Framing these actions through a One Health lens may also support greater interdisciplinary collaboration and funding, ensuring urban greening delivers cobenefits for human, animal, and environmental health (Mackenzie and Jeggo 2019). By approaching urban nature management through proactive design and planning, we can maximize the benefits of nature, mitigate the risks for it, and create resilient urban environments that work with rather than against it.

## Author biography

Sarah Bekessy (sarah.bekessy@unimelb.edu.au) is a professor at The University of Melbourne, Australia; Katherine Robertson (kmrobertson@student.unimelb.edu.au) is a PhD candidate at The University of Melbourne, Australia; and Jacinta Humphrey (jacinta.humphrey@rmit.edu.au) is a research fellow at RMIT University, Australia. All three conduct interdisciplinary research focused on solving complex biodiversity problems. Holly Kirk (holly.kirk@curtin.edu.au) is a senior research fellow at Curtin University, Australia, studying urban biodiversity conservation.
